# Mobile Data Collection: Smart, but Not (Yet) Smart Enough

**DOI:** 10.3389/fnins.2018.00971

**Published:** 2018-12-18

**Authors:** Alexander Seifert, Matthias Hofer, Mathias Allemand

**Affiliations:** ^1^University Research Priority Program “Dynamics of Healthy Aging”, University of Zurich, Zurich, Switzerland; ^2^Department of Communication and Media Research, University of Zurich, Zurich, Switzerland; ^3^Department of Psychology, University of Zurich, Zurich, Switzerland

**Keywords:** mobile data, experience sampling, real-life study, big data, smartphone

## Background

Mobile data collection with smartphones—which belongs to the methodological family of ambulatory assessment, ecological momentary assessment, and experience sampling—is a method for assessing and tracking people's ongoing thoughts, feelings, behaviors, or physiological processes in daily life using a smartphone (Mehl and Conner, 2012; Miller, 2012; Trull and Ebner-Priemer, 2013; Harari et al., 2016). The primary goal of this method is to collect in-the-moment or close-to-the-moment *active* data (i.e., subjective self-reports) and/or *passive* data (e.g., data collected from smartphone sensors) directly from people in their daily lives. The collection and assessment of such data is possible because smartphones are widely available and come with the computational power and sensors needed to obtain information about their owners' daily lives. Researchers in the fields of social science (e.g., Raento et al., 2009), psychology (e.g., Miller, 2012; Harari et al., 2016), and neuroscience (e.g., Schlee et al., 2016; Ladouce et al., 2017) use smartphones to collect data about personality processes and dynamics (Allemand and Mehl, 2017; Beierle et al., 2018a; Stieger et al., 2018; Zimmermann et al., 2018), daily cognitive behaviors (Aschwanden et al., 2018), social support behaviors (Scholz et al., 2016), momentary thoughts (Demiray et al., 2017), couple interactions (Horn et al., 2018), physical activity (Gruenenfelder-Steiger et al., 2017), and moods and emotions (Erbas et al., 2018).

Using smartphones for data collection provides a snapshot of individuals' everyday perceptions, experiences, and interactions with their environments. The use of mobile devices for the assessment of individuals' daily lives is not a new research method (e.g., Fahrenberg et al., 1996). However, because smartphones have now become so widespread throughout the population, are low in cost, and are equipped with sensor technology and ready for data collection through apps (Miller, 2012; Cartwright, 2016; Harari et al., 2016; Beierle et al., 2018a), we are now living in an interesting time for smart mobile data collection. Despite much progress, based on our experiences and discussions with experts in the field, we see the potential for further development of this method.

## Smart Mobile Data Collection

Mobile data collection with smartphones is growing rapidly in popularity due to its many advantages. One such advantage is that the findings are ecologically valid because they are collected during people's day-to-day lives and capture behaviors and experiences in real environments outside of research laboratories (Wrzus and Mehl, 2015). *Real-time* reports (i.e., active data) and sensor data (i.e., passive data) are measured in the moment and are therefore less prone to memory bias than are retrospective assessments (Redelmeier and Kahneman, 1996). By capturing real-time data about when and where an action takes place, the method provides important information about the dynamics of real-life patterns (Hektner et al., 2007). A smartphone allows researchers to capture such data by installing random, continuous, or event-based alarms to ask participants for their responses to questions or events during the day. Intensive repeated measurements of one participant capture *within-person* information, which represents the behaviors and experiences of a single individual. In contrast, *between-person* information demonstrates variability between individuals. Collecting within-person information allows for the study of the mechanisms and processes that underlie behavior, and this can be contrary to between-person information (Hamaker, 2012). For example, a study by Stawski et al. (2013) showed that processing speed is important for understanding between-person differences in working memory, whereas attention switching is of greater importance to within-person variations. Therefore, it can be argued that the proper study of the dynamic nature of psychological processes requires repeated observations within individuals (Conner et al., 2009). Smartphones are ideal tools for collecting such data.

Real-life data measurements are also rich in *contextual information*, as mobile data collection allows for the combination of self-reports or observer-reports (i.e., active data) and objective assessments (i.e., passive data) of activities, movements, social interactions, bodily functions, and biological markers, using the sensors that are built into smartphones (Ebner-Priemer et al., 2013). For example, it is possible to collect self-reports (e.g., individuals' feelings of social inclusion) and simultaneously to record acoustic sound clips of conversation to collect the objective patterns of participants' actual proximity to and interaction with others (e.g., Mehl et al., 2001).

Finally, as *measurement devices*, smartphones are both powerful and widespread in the population. This enables data analysis in real time and the opportunity to run machine learning approaches within the devices, allowing for large, individualized, dynamic, and intensive real-life studies (Raento et al., 2009; Bleidorn and Hopwood, 2018). Because most participants already have their own smartphones, an app is the only thing they need to install to participate in a study (Miller, 2012). This gives researchers the opportunity to conduct studies with large samples (Dufau et al., 2011).

## Smarter Mobile Data Collection in the Future

In our research, we identified some of the challenges accompanying mobile data collection with smartphones. In addition to discerning six challenging areas, we offer some suggestions for dealing with these challenges in the future. The first challenge relates to collecting data in real-life environments. Collecting smart data in daily life may result in the validation of existing theories, some of which may relate to behaviors and phenomena outside the realm of day-to-day life. However, this requires that researchers develop theories that reflect the multiple factors and dynamics of the real-life context that may influence the individual. Additionally, real-life data should not be collected simply because it is possible to do so, with conclusions about the theoretical significance of the data being drawn afterwards. Instead, we should develop and discuss the potential of real-life theories that consider both the within-person and between-person effects and the real-life context.

The second challenging area relates to real-time measurements. In data collection, *real-time* also means *right on time*; in other words, researchers have to carefully determine whether they are collecting data about the most relevant variables at the most appropriate moments and at ideal time intervals. To do so, they must first know when to collect data and when behaviors, thoughts, or changes are likely to occur. This question is crucial in mobile data collection, because conclusions about fluctuations, variability, and dynamics need to stem from a sound theoretical rationale or from the behavior patterns of the target participant (e.g., Wright and Hopwood, 2016). For instance, smartphone sensor technology and machine learning can help researchers by detecting the time points of events within a participant, by learning when events normally occur, or by learning the dependency of other subjective or objective variables upon events (e.g., Albert et al., 2012).

The third challenging area concerns within-person data. Typical smartphone studies collect data with great fidelity and generate large quantities of observations, placing the approach clearly within the domain of “big data” and requiring its associated advanced analytic techniques (Yarkoni, 2012; Fan et al., 2014). Working with big data requires highly technical expertise that researchers outside the field of computational science do not normally have. Resources must be organized, and after collecting the data, skills in advanced statistical analyses, including longitudinal structural equation modeling (Little, 2013), dynamic structural equation modeling (Asparouhov et al., 2017), multilevel modeling (Bolger and Laurenceau, 2013), and machine learning (e.g., Bleidorn and Hopwood, 2018), are required. As a result, an interdisciplinary research approach involving researchers interested in collecting data with smartphones and experts familiar with those forms of data collection, management, and analysis is crucial. Such endeavors should be supported by funding organizations and academic career programs, enabling the full potential of mobile data collection with smartphones to be achieved.

As a fourth challenging area, we identify the contextual information that can be collected with smartphone sensor data (i.e., passive data), as researchers have to consider the different forms, intervals, and amounts of sensor data (e.g., GPS data, app use, and accelerometer data). When collecting passive data continuously over multiple days, researchers need to consider more than just the data itself; they must also be able to interpret what the measurements indicate and convert the data into psychologically meaningful variables, such as sociability or mobility patterns (e.g., Mehl et al., 2006; Harari et al., 2016). Although this task is fundamental to the research, it often requires new skills of researchers and new approaches within the technology—approaches that ideally automatically aggregate passive smartphone-sensor-based data. For example, when collecting sound files containing conversation, it would be very helpful to automatically detect the spoken words of a target person (e.g., Mehl et al., 2001), detect contextual information (e.g., Lu et al., 2012), or interpret GPS data in terms of mobility patterns (e.g., Ryder et al., 2009). For such requirements, preliminary solutions do exist (e.g., Barry et al., 2006; White et al., 2011), but much more development and validation work is needed before we can achieve automatic, preprocessed, and validated smartphone-sensor data that can be combined with other types of data collection.

The fifth challenging area relates to the smartphone device itself. Mobile data collection with smartphones requires more technical preparation and greater technical confidence and skills, on the side of both the researcher and participant, than is required in classic paper-and-pencil studies. Daily technical hassles such as malfunctioning software and hardware, low smartphone batteries, and operation systems crashing during ongoing studies cost time and resources. Therefore, we highly recommend including an explicit time buffer and anticipating a higher than usual drop-out rate in smartphone studies to compensate for potential technical problems and challenges (for more information on technical issues, please see Mehl and Conner, 2012; Miller, 2012; Harari et al., 2016). Although the technical side of mobile data collection with smartphones is likely to become more reliable over time, more validation studies are required in this area and more ready-made valid apps are needed. When using smartphones for data collection within specific population groups, it is also important to consider the unique needs of the target group. For example, when working with older adults, it can be helpful to reflect participants' potential lack of smartphone skills by adapting briefings on smartphone/app use (Seifert et al., 2017).

The final, though certainly not least important, challenging area is that of data security and ethical issues. Collecting mobile data has revived past concerns about data protection and the ethical use of data. Using mobile devices for data collection, including tracking behavior and lifestyle patterns, introduces a unique dimension to individual participant protection. When collecting intensive profiles of individuals, which is the main research method within mobile data collection with smartphones, anonymization is nearly impossible. Therefore, traceable real-life data requires an intensive consideration of ethical and legal approval, the safeguarding of participant privacy, and the establishment of data security and data privacy (Harari et al., 2016; Marelli and Testa, 2018). As an example, Beierle et al. (2018b) conceived a privacy model for mobile data collection apps. Zook et al. (2017) present ten simple rules for responsible big data research, concluding that ethical and data protection issues should not prevent research but that it is vital to ensure “that the work is sound, accurate, and maximizes the good while minimizing harm” (Zook et al., 2017, p. 8). When using participants' own smartphones, it is also important that researchers acquire participants' consent to share self-recorded data with researchers (Gustarini et al., 2016). In a quantitative population survey among persons over 50 years of age, Seifert et al. (2018) found that more than the half of this demographic group is willing to share self-recorded data with researchers, regardless of participants' age, gender, education, technology affinity, or perceived health. The sharing and use of participants' own self-recorded data may require new models of participant involvement, with the goal of creating a trusted relationship between the data providers and researchers working with the data (Beierle et al., 2018b; Seifert et al., 2018).

## Conclusions

Mobile data collection with smartphones offers unique and innovative opportunities for studying human beings and processes in real life and real time. This approach offers researchers the opportunity to collect real-time reports of participants in their natural environment and within their individual dynamics and life contexts with the help of a regular smartphone. However, the approach also brings many challenges that provide interesting avenues for future developments. To date, mobile data collection with smartphones is already very smart, but we see the potential for even smarter mobile data collection in the future.

## Author Contributions

All authors worked on this paper from conception to final approval and share the same opinion.

### Conflict of Interest Statement

The authors declare that the research was conducted in the absence of any commercial or financial relationships that could be construed as a potential conflict of interest. The handling editor declared a past co-authorship with the authors.
